# Psychometric properties of the Sport Anxiety Scale-2 for Chinese adolescent athletes taking the National Sports College Entrance Examination

**DOI:** 10.3389/fped.2023.1161842

**Published:** 2023-10-23

**Authors:** Shichen Li, Changfa Tang, Cheng Guo, Te Bu

**Affiliations:** ^1^ College of Physical Education, Hunan Normal University, Changsha, China; ^2^ Hunan Provincial Research Base for Public Service of Sports, College of Physical Education, Hunan Normal University, Changsha, China; ^3^ Key Laboratory of Physical Fitness and Exercise Rehabilitation of Hunan Province, College of Physical Education, Hunan Normal University, Changsha, China

**Keywords:** trait anxiety, competition anxiety, factor structure, factorial invariance, gaokao

## Abstract

**Background:**

This study aims to evaluate the psychometric properties of the Sport Anxiety Scale-2 (SAS-2) in Chinese adolescent sports exam candidates.

**Methods:**

One day before the National Sports College Entrance Examination, 965 Chinese athletes rated the Chinese-language SAS-2. Confirmatory factor analysis was performed to test the three-factor structure. Factorial invariance was tested by comparing the configural invariance model to three more constrained models. Construct validity and reliability were determined.

**Results:**

Fit indices meet the critical values: CFI = 0.953, TLI = 0.943, RMSEA = 0.048 [90% CI, 0.041–0.054], and SRMR = 0.042. All path factor loadings exceed 0.5. The changes in CFI and RMSEA across the configural, metric, scalar, and uniqueness invariance are within the critical values, demonstrating strict measurement invariance across gender, years of training, and type of sports. The average variance extracted of the worry sub-scale is above the cutoff criteria, and McDonald's omega coefficients are over 0.70. Significant correlations exist between the SAS-2, SCAT, and CSAI-2. Factor correlations are all below 0.8. The measurement also distinguishes the known gender effect, with females showing a probability of 58.6% higher anxiety levels. The intraclass correlation coefficient ranges from 0.706 to 0.801.

**Conclusion:**

This study validated the Chinese-language SAS-2 in measuring competitive anxiety among Chinese adolescent athletes taking the National Sports College Entrance Examination. The development of the scale's applicability in China is discussed.

## 1. Introduction

While physical activity and exercise participation are generally enjoyable, sports competition is an extraordinarily stressful event for athletes since they have a limited chance to demonstrate their mastery of physical abilities, skills, and tactics to win the game (1). Athletes and sports psychologists are well aware that optimal performance extends beyond the mere result of physical training, and is often impacted by additional stress sources. Competitive anxiety is one such stressor that affects elite sports performance. Competitive anxiety is a sport-specific anxiety that frequently manifests (weeks) before or during the competition (2). Theoretical development has further specified competitive anxiety into somatic and cognitive dimensions (3). Signs of competitive anxiety include a negative, unpleasant emotional response to competition-related stressors, which can manifest as feelings of apprehension and tension (4) and cause increased levels of somatic arousal, worry, and/or concentration disruption during competitions (5). Athletes with elevated anxiety could result in sub-optimal sports performance (6).

It is important to distinguish between the trait and state dimensions of competitive anxiety. Competitive trait anxiety is a relatively stable personality trait, whereas competitive state anxiety is a transient state of mind on game day (7). Using Smith's original Sport Anxiety Scale, Hanton and colleagues examined the trait-state relationship among collegiate-level soccer players (8). It was found that those with high-trait anxiety exhibited higher levels of state anxiety than those with low-trait anxiety. Targeting competitive trait anxiety could therefore provide an early intervention opportunity for stabilizing game day performance.

Adolescence is the crucial period for the development of personality traits and mental health (9), as well as physical ability and motor skills (10). During this stage, adolescent athletes must not only contend with academic pressure and potential social and familial issues but also practice and compete in sports. Multiple stressors make adolescent athletes more susceptible to anxiety, and in the context of sports, heightened competitive anxiety has been associated with lower competitive levels, lack of motivation, and poorer sports performance (11). For Chinese adolescent athletes, this could have implications far beyond their athletic careers.

In historical Chinese society, the Sui Dynasty (581–618 A.D.) instituted the first national-level examination system to elect civil servants, which has now evolved into New China's National College Entrance Examination. Since 2019, more than 10 million Chinese high school students have taken the National College Entrance Examination annually, with test performance and university entrance directly influencing employment opportunities and long-term social prospects (12). While the National College Entrance Examination is considered a highly competitive public examination, enrollment in China's limited sports universities is even more challenging. For instance, between 2019 and 2022, the National College Entrance Examination admission rate in Hunan province was 36.89%, 45.24%, 45.40%, and 44.73%. During the same period, the National Sports College Entrance Examination admission rate in Hunan province was 17.97%, 17.91%, 24.32%, and 20.19%. In the event that a student-athlete does not meet the minimal admission score required by a sports university, they may face an uncertain professional trajectory as a physical education teacher, a trending occupation due to a groundbreaking shift in national policy (13).

Among the few published research on the anxiety levels of student-athletes taking the National Sports College Entrance Examination, student-athletes experience high levels of competitive anxiety. Specifically, 39.0%, 20.6%, and 11.9% of 400 student-athletes from Southwest China reported trait anxiety levels that were 2-, 3-, and 4-standard deviations higher than the general population, respectively (14). Similarly, among 127 student-athletes who took the 2012 National Sports College Entrance Examination, anxiety levels spiked one day before the examination (15). Wang and Zhang sampled 253 student-athletes in the 2006 National Sports College Entrance Examination (16). The 100-m run and shot put tests, both of which are commonly administered on the National Sports College Entrance Examination were affected among those who reported higher levels of anxiety as measured by the State-Trait Anxiety Inventory scale.

Although competitive anxiety during the National Sports College Entrance Exam is a known characteristic, research on this young cohort is exceedingly limited. The majority of sports psychology research conducted in China focuses on college, adult, and elite-level athletes. A major barrier to conducting relevant research is the lack of a Chinese-validated instrument to measure competitive anxiety in children and adolescents. For instance, previous research had to rely on the Hospital Anxiety and Depression Scale designed for clinical populations to measure sport-specific anxiety in adolescent athletes (17).

Several candidate scales exist for measuring competitive anxiety in Chinese adolescent athletes, such as the Sport Competition Anxiety Test (SCAT) (2) and the Competitive State Anxiety Inventory-2 (CSAI-2) (18). The SCAT is a uni-dimensional measure that does not differentiate between somatic and cognitive anxiety. Therefore, its utility for examining the cognitive dimension of competitive anxiety is constrained (3). The CSAI-2 is valid for assessing competitive state anxiety; however, it does not satisfy the overall objective of assessing competitive trait anxiety and intervening accordingly before the National Sports College Entrance Exam.

The Sport Anxiety Scale-2 (SAS-2) was developed as an instrument for assessing competitive anxiety in adults and children older than 9 years (19). The 15-item SAS-2 measures three dimensions of competitive trait anxiety, including cognitive anxiety (worry and concentration disruption) and somatic anxiety. Since its debut, the scale has attracted considerable interest and been validated in non-English contexts, including Brazil (20), Indonesia (21), Korea (22), Malaysia (23), Poland (24), and Spain (25).

The psychometric properties of the SAS-2 have not been validated in Chinese adolescent athletes. This study aimed to assess the construct validity of the SAS-2 among student-athletes taking the National Sports College Entrance Exam. In Chinese context, a valid and easy-to-use instrument for assessing adolescent competitive anxiety could have major societal implications.

## 2. Methods

### 2.1. Participants

This study was approved by the Institutional Review Board of the Hunan Normal University and conducted following the Helsinki Declaration. All student-athletes voluntarily participated, and adult participants or their legal guardians for minors provided signed informed consent.

High school student-athletes who took the 2019 National Sports College Entrance Examination in the Hunan province were contacted to participate in the study. The inclusion criteria required participants to take their first national exam and to be able to read and comprehend the test material. 965 student-athletes (including 255 females) participated in the study. Their mean (SD) age and training experience were 17.6 (0.5) years and 2.6 (1.1) years, respectively. Among them, 160 athletes took the ball sports exam, 124 for gymnastics, 248 for martial arts, and 433 for track and field.

### 2.2. Language adaptation

The SAS-2 measures 15 items across three dimensions of anxiety. On a 4-point Likert scale, respondents are requested to rate their somatic and cognitive anxiety (1 = “Not at all”, 2 = “A little bit”, 3 = “Pretty much”, and 4 = “Very much”). Individual item scores are added for each sub-scale score. Higher scores indicate a greater likelihood of competitive anxiety (19).

The language adaptation was carried out using typical translation-back translation processes in the field of psychology (26). Briefly, a Chinese-English bilingual translator translated the SAS-2 into Chinese, and another Chinese-English bilingual translator without access to the SAS-2 finished the back translation. The SAS-2 and the back-translated version were compared to keep the connotations of each item, and any discrepancies were reviewed between the two professional translators to reach an agreement. The first author, SL, who has more than 10 years of research expertise in sport psychology, examined the translation-back translation for face validity. Table 1 presents the Chinese-language SAS-2.

**Table 1:** Chinese-language sport anxiety scale-2 (SAS-2).

** **	Original SAS-2	运动焦虑量表-2				
** **	Guidance in English	Guidance in Chinese	Scale in Chinese			
	Please read each question. Then, circle the number that says how you USUALLY feel before or while you compete in sports. There are no right or wrong answers. Please be as truthful as you can.	以下的问题是有关你平时在运动竞赛中的感受。请在最接近你自己真实感觉的选项上打√	一点也不	有一点	有些	非常多
1.	It is hard to concentrate on the game	我很难把注意力集中在比赛上	1	2	3	4
2.	My body feels tense	我的身体紧绷	1	2	3	4
3.	I worry that I won't play well	我担心我不能取得好的成绩	1	2	3	4
4.	It is hard for me to focus on what I am supposed to do	我很难专注于我应该专注的事物 (比赛中的)	1	2	3	4
5.	I worry that I will let others down	我担心我会让其他人失望	1	2	3	4
6.	I feel tense in my stomach	我觉得胃发紧	1	2	3	4
7.	I lose focus on the game	在比赛中我走神了	1	2	3	4
8.	I worry that I will not play my best	我担心不能发挥出自己最好的水平	1	2	3	4
9.	I worry that I will play badly	我担心我会表现得很糟糕	1	2	3	4
10.	My muscles feel shaky	我感到肌肉在颤抖	1	2	3	4
11.	I worry that I will mess up during the game	我担心我在比赛时会犯错误	1	2	3	4
12.	My stomach feels upset	我觉得胃不舒服	1	2	3	4
13.	I cannot think clearly during the game	在比赛中我不能清晰地思考	1	2	3	4
14.	My muscles feel tight because I am nervous	我感到紧张所以我的肌肉紧绷	1	2	3	4
15.	I have a hard time focusing on what my coach tells me to do	我很难集中精力做教练让我做的事情	1	2	3	4

### 2.3. Instrument

The SCAT (2) and CSAI-2 (18) were used to evaluate the convergent validity of the Chinese-language SAS-2. The SCAT consists of 15 items using a 3-point Likert scale (1 = “Rarely”, 2 = “Sometimes”, and 3 = “Often”). 10 items measure anxiety-related symptoms and 5 items are included to reduce response bias and are therefore not scored. A total score is calculated by adding the 10 items, and a higher score indicates a greater likelihood of competitive anxiety. This study applied the Chinese-language SCAT, which has been validated in 10 to 25-year-old Chinese athletes (27). McDonald's omega for the Chinese-language SCAT in this study was 0.708.

The CSAI-2 consists of 27 items measuring cognitive state anxiety, somatic anxiety, and self-confidence on a 4-point Likert scale (1 = “Not at all”, 2 = “Somewhat”, 3 = “Moderately so”, and 4 = “Very much so”). This study applied the Chinese-language CSAI-2, which has been validated in 10 to 30-year-old Chinese athletes participating in ball sports, gymnastics, martial arts, and other sports (28). In the present study, McDonald's omega for the cognitive state anxiety, somatic anxiety, and self-confidence of the Chinese-language CSAI-2 were 0.842, 0.781, and 0.797, respectively.

### 2.4. Procedures

Because of the large number of exam candidates, the 2019 National Sports College Entrance Examination was administered in the Hunan province from April 10 to April 24. Exam candidates were divided into five groups and participated in a three-day exam: the first day was for registration, and the second and third days were examination days. On the day of registration, researchers contacted exam candidates at the registration center and presented participation invitations. After signing the informed consent, participants were asked to complete a survey regarding their age, gender, and type of sports. Participants then completed the Chinese-language SAS-2, SCAT, and CSAI-2 in random order. 1,000 exam candidates were invited, and 965 valid questionnaires were gathered. Research assistants were in the field at all times to address inquiries about research. Four weeks after the initial evaluation, 125 participants were chosen at random from 965 student-athletes to retake the Chinese-language SAS-2 at the Hunan Normal University.

### 2.5. Statistics

IBM SPSS version 22 and Mplus version 7.0 were used for statistical analyses. In this study, a two-tailed *p-*value of less than 0.05 is considered statistically significant, and the Bonferroni adjustment was applied to the Kruskal-Wallis *H* statistics to account for multiple comparisons.

Confirmatory factor analysis was conducted to examine the model fit. Distributions of scale scores were positively skewed in the original SAS-2 (19) and other language versions (25). In the present study, the normalized quantile residuals and Cramér–von Mises statistic (Table 2) confirm that none of the Chinese-language SAS-2 sub-scales conform to a normal distribution. To deal with the data structure, a robust maximum likelihood estimation was used in the confirmatory factor analysis. We calculated the ratio of chi-square statistic to degrees of freedom (*χ*^2^/df), comparative fit index (CFI), Tucker-Lewis index (TLI), root mean square error of approximation (RSMEA), and standard root mean square residual (SRMR). The following cutoff criteria were chosen to ensure a proper fit: CFI, >0.90 as marginal, ≥0.95 as excellent (29); TLI, >0.90 as acceptable, ≥0.95 as good (30); RMSEA, ≤0.08 as adequate, <0.05 as good (30); or, SRMR, ≤0.08 as acceptable (29). Because the *χ*^2^ is too sensitive to sample size and assumes the variables are multivariate normal, this metric may be treated as descriptive and not diagnostic of model fit (31). Nevertheless, we refer to the *χ*^2^/df ratio between 0 and 2 as a good fit and between 2 and 3 as an acceptable fit (32). If the model is acceptable, the factor loadings for each SAS-2 item are examined (29). Standardized regression coefficients (i.e., factor loadings) were calculated for each SAS-2 item, with values equal to or greater than 0.50 indicating practically significant (33).

**Table 2:** Summary of normalized quantile residuals and cramér–von mises *p*-values.

Subgroup	*N*	Somatic anxiety			Worry			Concentration disruption		
		Skewness	Kurtosis	*p*	Skewness	Kurtosis	*p*	Skewness	Kurtosis	*p*
Gender										
Male	710	0.538	2.734	<0.001	0.228	2.510	<0.001	0.491	2.837	<0.001
Female	255	0.601	2.764	<0.001	0.191	2.598	0.001	0.501	2.675	<0.001
Years of training										
<3	423	0.590	2.731	<0.001	0.189	2.631	<0.001	0.538	3.093	<0.001
≥3	542	0.539	2.780	<0.001	0.210	2.458	<0.001	0.487	2.638	<0.001
Type of sports										
Ball sports	160	0.698	3.319	<0.001	0.027	2.412	0.012	0.353	2.234	<0.001
Gymnastics	124	0.559	2.362	<0.001	0.016	2.148	0.012	0.146	2.730	0.012
Martial arts	248	0.501	3.070	<0.001	0.132	2.606	0.002	0.476	2.744	<0.001
Track and field	433	0.547	2.547	<0.001	0.330	2.615	<0.001	0.706	3.176	<0.001
Baseline	965	0.561	2.763	<0.001	0.201	2.537	<0.001	0.511	2.832	<0.001

Factorial invariance refers to the invariance of a factor model across different groups, and a valid model is expected to demonstrate consistency among measurement groups (34). Multi-group confirmatory factor analysis was conducted to assess the measurement invariance of the Chinese-language SAS-2 across gender (male vs. female), years of training (<3 years vs. ≥3 years), and type of sports (ball sports vs. gymnastic vs. martial arts vs. track and field), with male, less than 3 years of training, and track and field athletes serving as the Mplus reference groups. Specifically, the measurement invariance was conducted for increasingly restrictive levels of invariance: the configural invariance (the pattern of free and fixed model parameters), metric invariance (equal factor loadings across groups), scalar invariance (equal item intercepts across groups), and uniqueness invariance (equal item error variances/covariances across groups) (35). The following cutoff criteria were accepted as measurement invariance for unequal sample sizes in this study: change in CFI, <-0.005; change in RMSEA, <0.010; or, change in SRMR, <0.025 for testing the metric invariance, <0.005 for testing the scalar or uniqueness invariance (36).

The average variance extracted (AVE) (37) and McDonald's omega were calculated associated with convergence and reliability. Acceptable levels are indicated by sub-scales with an AVE of 0.5 or higher and/or McDonald's omega greater than 0.7 (33). In the meantime, the correlation was used to assess the convergent validity in comparison to other established instruments. The Spearman's rank correlation coefficients between the SAS-2, SCAT, and CSAI-2 were examined, and the values were interpreted based on the following cutoff criteria (38): *ρ* = 0–0.1 corresponds to negligible correlation; *ρ* = 0.1–0.39 corresponds to weak correlation; *ρ* = 0.4–0.69 corresponds to moderate correlation; *ρ* = 0.7–0.89 corresponds to strong correlation; and, *ρ* = 0.9–1 corresponds to very strong correlation. Rönkkö and Cho's factor correlation method (39) was used to test the discriminate validity. In brief, the latent variables were scaled by fixing their variances to one. When both the point estimate and its 95% upper limit are less than 0.8 and the likelihood ratio tests are significant (our constrained model used a cutoff of 0.8), this provides evidence of discriminate validity. These calculations were performed using the R packages lavaan version 0.6-16 and semTools version 0.5-6 in the RStudio version 2023.03.1 Build 446. For the known-group validity, the Mann–Whitney and Kruskal–Wallis *H* statistics were used to compare the scores from the three sub-scales across subgroups. For significant subgroup differences, we calculated the non-parametric, probability-based effect size *A* statistic (40). The calculation was performed using the R package dplyr version 1.1.2.

To determine the reliability of the Chinese-language SAS-2, we calculated the intraclass correlation coefficient (ICC) for test-retest reliability. The following critical values were applied for diagnostics: ICC < 0.5 corresponds to poor; ICC = 0.5–0.75 corresponds to moderate; ICC = 0.75–0.9 corresponds to good; and ICC > 0.9 corresponds to excellent (41).

## 3. Results

### 3.1. Factor structure

**Figure 1:**
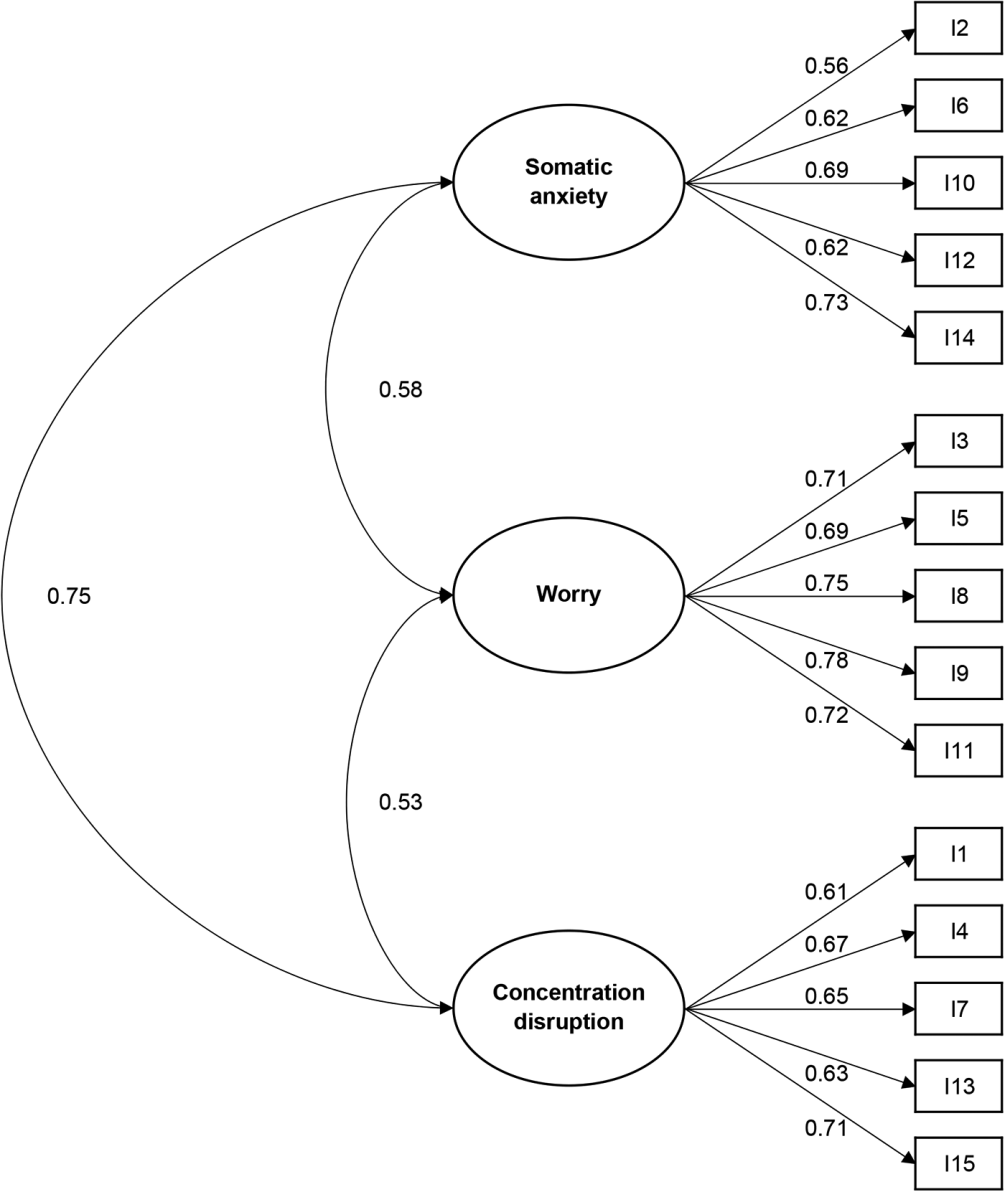
Standardized item-scale factor loadings of the Chinese-language sport anxiety scale-2 (*N* = 965). The values in the figure are standardized coefficients.

Table 3 lists fit indices for the Chinese-language SAS-2, demonstrating acceptable model fit. Table 4 summarizes goodness-of-fit metrics for other SAS-2 language versions. In general, the psychometric properties in this study are comparable to the original and other language adaptations of the SAS-2. Figure 1 shows the path diagram of the three-factor model. All item-factor path loadings exceed 0.5, and six item-factor path loadings exceed 0.7. In addition, the scale-factor path loadings connecting somatic anxiety, worry, and concentration disruption all exceed 0.5.

**Table 3:** Fit indices of the Chinese-language sport anxiety scale-2.

Subgroup	*χ*^2^/df	CFI	TLI	RMSEA [90% CI]	SRMR
Gender					
Male	2.60	0.951	0.941	0.047 [0.040, 0.055]	0.044
Female	1.81	0.943	0.932	0.056 [0.042, 0.070]	0.055
Years of training					
<3	2.24	0.940	0.928	0.054 [0.044, 0.064]	0.052
≥3	2.04	0.960	0.952	0.044 [0.034, 0.053]	0.042
Type of sports					
Ball sports	1.21	0.975	0.970	0.036 [0.000, 0.059]	0.052
Gymnastics	1.19	0.974	0.968	0.039 [0.000, 0.065]	0.054
Martial arts	1.74	0.930	0.916	0.055 [0.040, 0.069]	0.054
Track and field	2.01	0.953	0.943	0.048 [0.038, 0.059]	0.051
Baseline	3.19	0.953	0.943	0.048 [0.041, 0.054]	0.042

CFI, comparative fit index; RSMEA, root mean square error of approximation; SRMR, standardized root mean square residual; TLI, Tucker-Lewis index; *χ*^2^/df, ratio of chi-square statistic to degrees of freedom (under robust maximum likelihood estimation). The goodness of fit is accepted if: CFI, >0.90 as marginal fit, ≥0.95 as excellent fit; TLI, >0.90 as acceptable fit, ≥0.95 as good fit; RMSEA, ≤0.08 as adequate, <0.05 as good fit; SRMR, ≤0.08 as acceptable fit; or, *χ*^2^/df, ≤2 as good fit, ≤3 as acceptable fit.

**Table 4:** Language-adapted versions of the sport anxiety scale-2.

Language (citation)	*N*	Female (%)	Age (years)	Goodness of fit	
				CFI	RMSEA
English (19)	850	-	9–14	0.96	0.05
English (19)	1,038	45.0	9–14	-	-
Chinese (Li et al., current)	965	26.4	17–18	0.95	0.05
Indonesian (21)	268	42.5	16–43	0.92	0.08
Korean (22)	303	34.7	19–25	0.92	0.07
Malay (23)	457	30.0	8–27	0.93	0.06
Polish (24)	519	48.7	22.83	0.95	0.07
Portuguese (20)	238	29.0	13–53	0.97	0.08

CFI, comparative fit index; RSMEA, root mean square error of approximation.

### 3.2. Measurement invariance

Table 5 summarizes the configural invariance, metric invariance, scalar invariance, and uniqueness invariance that were tested across subgroups. The differences in unconstrained and constrained models were within thresholds, thus indicating that factor loadings, intercepts, and residual variances were invariant across gender, years of training, and type of sports (except for ΔSRMR of the residual variances).

**Table 5:** Measurement invariance of the Chinese-language sport anxiety scale-2.

Subgroup	Comparison	ΔCFI	ΔRMSEA	ΔSRMR
Gender				
Configural invariance (*I_C_*)	–	–	–	–
Metric invariance (*I_M_*)	*I_M_*−*I_C_*	−0.002	−0.001	0.004
Scalar invariance (*I_S_*)	*I_S_*−*I_M_*	0.000	−0.001	0.001
Uniqueness invariance (*I_U_*)	*I_U_*−*I_S_*	−0.002	−0.001	0.002
Years of training				
Configural invariance (*I_C_*)	–	–	–	–
Metric invariance (*I_M_*)	*I_M_*−*I_C_*	−0.001	−0.002	0.003
Scalar invariance (*I_S_*)	*I_S_*−*I_M_*	−0.002	0.000	0.000
Uniqueness invariance (*I_U_*)	*I_U_*−*I_S_*	−0.001	−0.001	0.002
Type of sports				
Configural invariance (*I_C_*)	–	–	–	–
Metric invariance (*I_M_*)	*I_M_*−*I_C_*	−0.001	−0.001	0.007
Scalar invariance (*I_S_*)	*I_S_*−*I_M_*	0.000	−0.002	0.000
Uniqueness invariance (*I_U_*)	*I_U_*−*I_S_*	−0.003	−0.001	0.006

CFI, comparative fit index; RSMEA, root mean square error of approximation; SRMR, standardized root mean square residual. Measurement invariance is accepted if: ΔCFI, <-0.005; ΔRMSEA, <0.010; or, ΔSRMR, <0.025 for testing metric invariance, <0.005 for testing scalar or uniqueness invariance.

### 3.3. Convergent validity

The AVE estimates for somatic anxiety, worry, and concentration disruption were 0.417, 0.528, and 0.428, respectively. The McDonald's omega coefficients for somatic anxiety, worry, and concentration disruption were 0.780, 0.848, and 0.789, respectively, which were all higher than the guideline value.

Table 6 summarizes correlations between competitive anxiety scales. There were moderate correlations between the Chinese-language SAS-2 and the SCAT. In addition, moderate, positive correlations existed between the Chinese-language SAS-2 and the somatic anxiety and cognitive state anxiety of the CSAI-2. As expected, there were weak, negative correlations between the Chinese-language SAS-2 and the self-confidence of the CSAI-2.

**Table 6:** Between-scale Spearman's correlation coefficients.

Scale	1	2	3
1. SAS-2: Somatic anxiety	–	–	–
2. SAS-2: Worry	0.471**	–	–
3. SAS-2: Concentration disruption	0.611**	0.459**	–
4. SCAT	0.518**	0.517**	0.480**
5. CSAI-2: Somatic anxiety	0.408**	0.542**	0.421**
6. CSAI-2: Cognitive state anxiety	0.536**	0.400**	0.454**
7. CSAI-2: Self-confidence	-0.206**	-0.237**	-0.289**

CSAI-2, Competitive State Anxiety Inventory-2 (Chinese language); SAS-2, Sport Anxiety Scale-2 (Chinese language); SCAT, Sport Competition Anxiety Test (Chinese language).

** *p* < 0.01.

### 3.4. Discriminate validity

Table 7 presents the factor correlation analysis. The point estimate and its 95% upper limit of every factor pair fall within the cutoff point, and the *χ*^2^ statistics are all significant, suggesting the Chinese-language SAS-2 achieves discriminate validity.

**Table 7:** Estimated factor correlation.

Pair	CI_CFA_ (sys)	*χ*^2^ (sys)		
	Upper 95%	*ρ*_CFA_	*χ*^2^ difference	*p* (*χ*^2^)
Somatic anxiety—Worry	0.635	0.579	88.8	<0.001
Somatic anxiety—Concentration disruption	0.799	0.753	4.4	0.037
Worry—Concentration disruption	0.592	0.532	125.8	<0.001

### 3.5. Known-groups validity

**Figure 2:**
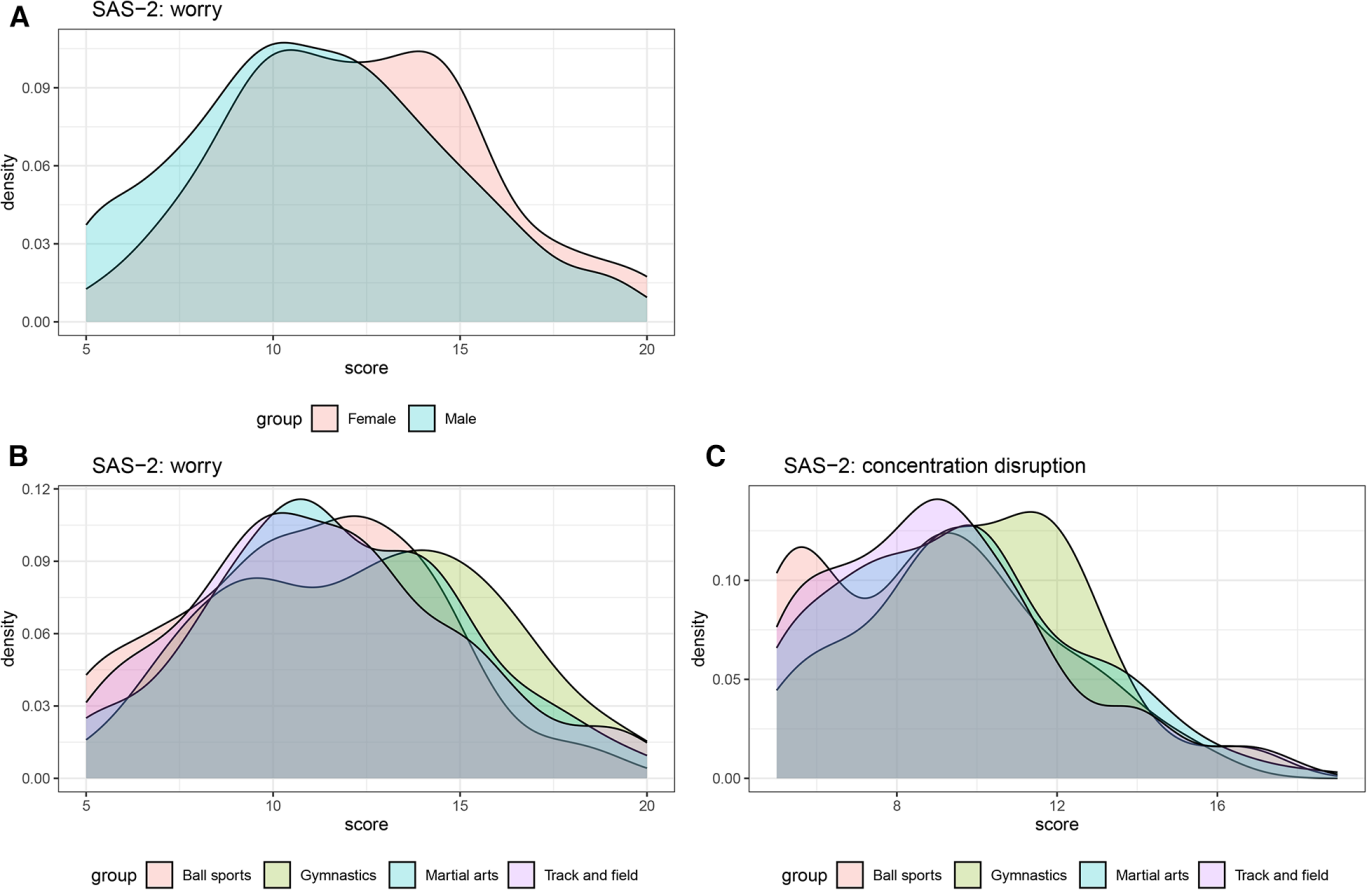
Densities of the Chinese-language sport anxiety scale-2 for each factor level. (**A**) Gender difference in the worry sub-scale; (**B**) type of sports difference in the worry sub-scale; (**C**) type of sports difference in the concentration disruption sub-scale.

As shown in Table 8 and Figure 2, gender and type of sports are associated with anxiety levels. Calculating the *A* statistic yields a value of 0.586, indicating a 58.6% probability that a randomly chosen woman will have higher anxiety levels on the worry sub-scale than a randomly chosen man. On the worry sub-scale, there is a 61.2% and a 58.0% probability that a random gymnast will have higher anxiety levels than athletes from ball sports and track and field, respectively. On the concentration disruption sub-scale, there is a 61.7% and 60.1% probability that a random gymnast will have higher anxiety levels than athletes from ball sports and track and field, respectively.

**Table 8:** Between-group comparisons of the Chinese-language sport anxiety scale-2.

Sub-group	Somatic anxiety		Worry		Concentration disruption		Total score	
	*M* (SD)	*p*	*M* (SD)	*p*	*M* (SD)	*p*	*M* (SD)	*p*
Gender								
Male	9.04 (2.79)	0.706	11.22 (3.51)	<0.001	9.21 (2.87)	0.074	29.46 (7.51)	0.007
Female	9.16 (2.96)		12.27 (3.36)		9.67 (3.16)		31.11 (7.62)	
Years of training								
<3	8.92 (2.82)	0.138	11.50 (3.47)	0.895	9.25 (2.90)	0.617	29.67 (7.47)	0.553
≥3	9.19 (2.85)		11.49 (3.52)		9.39 (3.00)		30.07 (7.65)	
Type of sports								
Ball sports	8.77 (2.80)	0.263	10.91 (3.33)	0.008	8.88 (2.92)	0.002	28.56 (7.62)	0.007
Gymnastics	9.08 (3.13)		12.35 (3.59)		10.05 (2.77)		31.48 (7.67)	
Martial arts	9.26 (2.66)		11.67 (3.38)		9.50 (3.00)		30.43 (7.19)	
Track and field	9.07 (2.86)		11.37 (3.56)		9.19 (2.96)		29.63 (7.65)	

### 3.6. Reliability

ICC values for somatic anxiety, worry, and concentration disruption were 0.764, 0.706, and 0.747, respectively. Thus, the Chinese-language SAS-2 has moderate to good test-retest reliability.

## 4. Discussion

In modern competitive sports, sports psychology has become an integral part of the preparation cycle for major competitions to strengthen the psychological control ability of elite athletes and enable them to perform at least at their normal training level during the tournament (42). Psychological intervention has always been one of China's essential scientific tools for ensuring its Olympic success (43). This scientifically proven field has not, however, been incorporated into the preparation of the National Sports College Entrance Examination. Given the low enrollment rate at Chinese sports universities, student-athletes’ future careers may be hindered by competitive anxiety during the one-shot competition-style examination. To address this practical need, this study validated a youth-appropriate competitive anxiety scale.

For goodness-of-fit statistics in structural equation model analysis, several fit indices have been proposed (30); though there is no consensus on one particular golden criterion. Common criteria in this field of study include the *χ*^2^ (23), CFI (25), RMSEA (19), and SRMR (24). In addition, various cutoff points (30) add an additional layer of complexity to the objective interpretations of fit indices. This study adopted the popular Hu and Bentler's cutoff points (29) in SAS-2 research. Accordingly, the three-factor model generally fits the data well in terms of the CFI, TLI, RMSEA, and SRMR.

One exception is the *χ*^2^/df ratio. Based on the Schermelleh-Engel and colleagues’ criteria (32), the overall level of model fit does not meet the acceptable threshold, albeit marginally (observed 3.19 vs. recommended 3). The problem here is twofold. First, the *χ*^2^ is based on the assumption that observations are multivariate normal, which is not always met in this field of research (25) and the present study (Table 2). In the original paper evaluating the novel SAS-2 structural model, Grossbard and colleagues used logarithmic transformation to resolve the issue of non-normality in their data (5). To overcome this practical problem, this study applied the robust maximum likelihood estimation, which is supposed to resolve this estimation-method effect. Notwithstanding this correction, the accuracy of the *χ*^2^ has to be considered on top of another factor. Large sample size impacts the *χ*^2^, hence using the *χ*^2^ as a measure of model fit may result in an inflated Type I error rate for model rejection (44). This effect, in our opinion, explains why the *χ*^2^/df ratio behaves differently for various sample sizes in the present study. Specifically, the *χ*^2^/df ratios in all subgroup analyses meet Schermelleh-Engel's criteria, whereas the overall data are rejected, even though there is no discernible difference in the overall data structure. In fact, one of the original proponents of the *χ*^2^/df ratio later advised against its application (45).

Fairly speaking, these one-size-fits-all guidelines, such as Hu and Bentler's CFI of 0.95 or Schermelleh-Engel's CFI of 0.97 (32) as the acceptable threshold, are overly conservative for certain models in practice, and we are not the lone doubters (46). A recent theoretical advancement is Mai and colleagues’ tailored-fit model evaluation strategy (47) to bridge the various theories regarding goodness-of-fit statistics in applied research. Without taking into consideration different sample sizes, their simulation study suggests that the flexible CFI cutoffs for Gaussian, moderate non-Gaussian, and severe non-Gaussian data are 0.916, 0.904, and 0.880, respectively. Given that the CFI is sensitive to model misfit and does not depend on sample size as strongly as the *χ*^2^ (31), the CFI is an optimal indicator of model fit in the present study with its relatively large sample size and highly skewed data distribution. Using either classic or flexible cutoffs, the model fit passes the test statistics. In this sample, 14 of the 15 item-factor loadings exceed 0.6 and all path loadings exceed 0.5, demonstrating construct validity (33). These confirmatory factor analyses provide support for the factor structure of the SAS-2 among Chinese adolescent athletes.

The three-factor model exhibits structural consistency across subgroups. Specifically, the Chinese-language SAS-2 measured the same precision in 17- to 18-year-old male and female Chinese student-athletes, supporting gender invariance confirmed in Korea (22), Malaysia (23), Poland (24), Spain (25), and the USA (5). This study expanded the invariance of measurement concerning the training experience and sports participation. The unconstrained and constrained models showed comparable levels of fit, demonstrating that there is no construct bias in these two aspects. Notably, the measurement invariance is validated up to the level of uniqueness, indicating that subgroups have equal factor loadings, indicator intercepts, and error variances and covariances. Therefore, the Chinese-language SAS-2 establishes critical factorial invariance and broadens our understanding of the overall validity of the original SAS-2.

The AVE is employed as an indicator of convergent validity, and an acceptable AVE estimate is 0.5 or higher (33). In this study, only the worry sub-scale achieved the cutoff point. Nonetheless, this should not be a justification to exclude items from the somatic anxiety and concentration disruption sub-scales. Based on the reported factor loadings (see also their Figure 1) (19), we can calculate the AVE estimates for the original scale, which were 0.494, 0.574, and 0.456 for the somatic anxiety, worry, and concentration disruption, respectively. Therefore, the original SAS-2 did not meet the general AVE requirement. Neither the Indonesian-language SAS-2 (21) nor the Korean-language SAS-2 (22) met this specific requirement in its entirety. In this case, however, it is also agreed that a construct meets the requirement for convergent validity if the McDonald's omega is greater than 0.7 (33, 37). All McDonald's omega coefficients in this study were greater than 0.7. In addition, significant correlations were found between the SAS-2 and the trait-anxiety SCAT and state-anxiety CSAI-2, and the factor correlation analysis further supports the discriminate validity. Collectively, these results provide additional evidence for the construct validity of the Chinese-language SAS-2.

The measurement invariance reveals that the items of a construct were measured with the same precision across subgroups, whereas group variations on any item are attributable solely to group differences in the common factors (35). The gender effect is a known construct when measuring competitive anxiety (11). In both the American (5) and European (25) populations, females scored higher on the SAS-2. In China, female candidates in the National Sports College Entrance Examination exhibited higher levels of competitive state anxiety than their male counterparts (15). Our findings that female adolescent athletes had higher anxiety levels, albeit not with a high probability, on the worry sub-scale of the SAS-2 back this known effect and provide additional evidence of its construct validity.

The current findings suggest that gymnasts may have an innately higher level of competitive anxiety. Early research indicates that 9- to 14-year-old males experience higher competitive state anxiety in individual sports like gymnastics than in team sports like basketball (48). This is also true of higher-level athletes (49). Similarly, golfers, who are distinguished by their fine motor skills, exhibited a higher level of competitive state anxiety than rugby players (50). Here, gymnasts had higher anxiety levels on the worry and concentration disruption sub-scales of the SAS-2. However, it should be mentioned that the gymnasts in this study are mostly females (84 out of the total 124), hence there is a scope that this sports-type effect may be influenced by the specific gender effect.

It is worth mentioning the ecological validity. In this study, student-athletes rated the SAS-2 on the day preceding the National Sports College Entrance Examination, a time when their anxiety level should be at its peak. The objective here is to isolate the effects of other kinds of daily stress on competitive anxiety, ensuring that the measured anxiety contained the least amount of noise for evaluating the psychometric properties of the SAS-2. In practice, student-athletes’ competitive trait anxiety should be assessed weeks or months before the National Sports College Entrance Examination. The SAS-2 is ultimately a helpful tool for interventional purposes. A sufficient lead time could let practitioners develop tailored strategies to assist student-athletes in better managing competitive anxiety and enhancing examination performance.

More than 10 million high school students take the National College Entrance Examination each year. Since the introduction of the “Opinions on Deepening the Integration of Sports and Education to Promote the Healthy Development of Adolescents” (51), sports examinations (e.g., 50-m run, standing long jump) are gradually becoming an integral part of the National College Entrance Examination, requiring students to take sports evaluation as part of their overall examination score for university admission. Students with little or no sports competition experience are presumably more prone to competitive anxiety (52), and extending SAS-2 to this cohort could have profound societal ramifications. To broaden and generalize the application of the Chinese-language SAS-2, research should be conducted on younger (14-year-olds enrolling in high school) and less athletically competent students. For future validation and intervention studies, it is worthwhile to investigate the criterion validity with objective measures of athlete performance.

Finally, any development of psychometric measures is incomplete without a straightforward interpretation of their practical significance in real-world circumstances. While a higher SAS-2 score indicates higher competitive anxiety, it is unclear how high a score must be to warrant intervention. Until an objective SAS-2 cutoff point or, even better, the population-based percentile is exhaustively determined, the Chinese-language SAS-2 concept remains theoretical.

In conclusion, this is the first study to confirm the psychometric properties of the SAS-2 in the Chinese context, which might be useful for nearly half a million Chinese adolescents who pursue a career in higher-level sports at universities each year. The three-factor model exhibits adequate fit, its factor structure remains strictly invariant across gender, training experience, and type of sports, and reliably measures the trait anxiety spanning a four-week, non-intervention interval. Theoretically, practitioners could use this Chinese-language SAS-2 to identify competitive anxiety in Chinese student-athletes between the ages of 17 and 18. These findings pave the way for a scientific evaluation of competitive anxiety and the development of mitigation strategies for the millions of Chinese high school students taking the National College Entrance Examination.

## Data Availability

The raw data supporting the conclusions of this article will be made available by the authors, without undue reservation.
